# Biomechanical stabilization of the keratoconic cornea: a review of current therapeutic strategies

**DOI:** 10.3389/fmedt.2026.1919880

**Published:** 2026-10-14

**Authors:** Graziana M. Ragonese, Edoardo Grosso, Dario Carbonaro, Elena Torta, Emiliano Lepore, Diego Gallo

**Affiliations:** 1 Polito^BIO^Med Lab, Department of Mechanical and Aerospace Engineering, Politecnico di Torino, Torino, Italy; 2 Recornea Srl, Trieste, Italy; 3 Department of Mechanical, Energetic and Management Engineering, Università della Calabria, Arcavacata di Rende, Italy

**Keywords:** collagen crosslinking, corneal biomechanics, ectatic cornea, intracorneal ring segments, keratoconus, visual rehabilitation

## Abstract

Keratoconus is a progressive corneal ectatic disorder characterized by stromal thinning and biomechanical weakening of the cornea, leading to progressive corneal protrusion and visual impairment. Therapeutic strategies have evolved from visual correction toward approaches specifically targeting the biomechanical instability underlying disease progression. This review provides a biomechanical perspective on current and emerging strategies for the biomechanical stabilization of the keratoconic cornea. Conservative treatments based on contact lenses remain essential for visual rehabilitation but do not modify the underlying ectatic process. Corneal collagen crosslinking represents the reference treatment for progressive keratoconus, as it increases stromal stiffness through the formation of additional covalent bonds. However, its efficacy depends on treatment protocol, epithelial status, corneal thickness, and disease stage. Corneal implants, i.e., synthetic intracorneal ring segments and corneal allogenic intrastromal ring segments, provide stretch-induced geometric improvement of the corneal shape and, consequently, of visual acuity, although predictability remains limited. Combined approaches, such as ring implantation followed by cross-linking, aim to integrate corneal reshaping with intrinsic stromal reinforcement. Emerging approaches, including cell-based therapies, and implantation of stromal lenticule or tissue-engineered scaffolds, suggest a future shift toward a multimodal therapeutic strategy integrating biomechanical stabilization, restoration of stromal structure, and biological regeneration. Advancing keratoconus management will require objective biomechanical assessment and predictive modeling tools to guide individualized treatment planning and improve outcome predictability. Ultimately, future management should increasingly incorporate a biomechanical perspective, with interventions designed to modify the mechanical behavior of the ectatic cornea, arrest progression, and restore functional corneal integrity.

## 1 Introduction

Keratoconus (KCN) is a progressive corneal ectasia characterized by stromal thinning (1) and biomechanical weakening, which reduces the ability of the cornea to maintain its physiological curvature under intraocular pressure (IOP). This leads to conical protrusion and consequent vision distortion (2). Corneal shape is maintained by the highly ordered organization of stromal collagen fibrils. In KCN, this organization is disrupted and has been associated with reduced natural collagen crosslinking, further impairing structural integrity (3). A recent global systematic review and meta-analysis estimated a prevalence of approximately 289 cases per 100,000 individuals (0.24%) and an incidence of 4.0 per 100,000 person-years, with markedly higher prevalence in Africa and South Asia (4). Disease onset typically occurs during the second decade of life with mild corneal thinning and slight refractive changes (5), and it may progress until the fourth decade, when corneal morphology generally stabilizes (6). As the ectasia advances, increasing corneal irregularity leads to visual disturbances such as glare, halos, photophobia, monocular diplopia, and progressive visual blurring (7). End-stage disease may range from mild refractive errors to severe astigmatism, myopia, or visual loss (8). As KCN worsens, surgical intervention becomes necessary in 15% to 20% of affected individuals (9)**.** Corneal transplantation (i.e., keratoplasty) has been the mainstay of surgical treatment for patients with advanced KCN. There is, however, a considerable shortage of corneal graft tissue, with only 1 human donor cornea available for 70 needed (10). Moreover, in about 10% of cases keratoplasty is associated with a long-term risk of immune-mediated endothelial rejection, endothelial cell loss, and complications such as expulsive hemorrhage and endophthalmitis (11).

Several procedures and implants have been developed to biomechanically stabilize the keratoconic cornea, thereby delaying or potentially avoiding the need for keratoplasty. These approaches range from external mechanical support, through intrinsic reinforcement of the corneal stroma, to structural implants that reshape and stabilize the cornea (Table 1). In the early stages of the disease, KCN management relies on conservative approaches, mainly spectacles and rigid contact lenses (CLs), which can provide satisfactory refractive and visual correction (12) but do not address the underlying biomechanical instability of the cornea. Corneal collagen crosslinking (CXL) was subsequently introduced as an early-stage therapeutic option to halt keratoconus progression by inducing additional cross-links within the collagen matrix and thus increasing corneal biomechanical stiffness (13). In recent years, minimally invasive surgical options such as intracorneal ring segments (ICRSs) made of polymethyl methacrylate (PMMA) or derived from allogenic tissue (corneal allogenic intrastromal ring segments, CAIRS) have also been proposed (14). These approaches act on the corneal periphery to induce central corneal flattening, thereby reducing corneal steepening in keratoconic eyes.

**Table 1:** Comparative overview of established and emerging approaches for keratoconus management.

	Approach	Mechanism of Action	Biomechanical Impact	Clinical Outcomes	Challenges/Limitations	Biomechanical Reinforcement	Geometrical Modification
Established approaches	Contact Lenses (CLs)	Formation of a tear lens between the CL and the cornea that masks corneal irregularity and improves the optical surface	Possible external mechanical support, depending on CL design and fitting; no intrinsic stromal reinforcement	Improved visual quality and reduction of irregular astigmatism in mild disease	No modification of the underlying ectatic process; intolerance or poor fitting in advanced disease; risk of epithelial damage or corneal thinning if improperly fitted	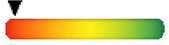 Very low: external support only	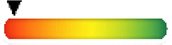 Very low: no direct reshaping
	Corneal Collagen Cross-Linking (CXL)	Riboflavin/UV-A-induced formation of additional covalent bonds within the stromal collagen matrix	Direct increase in stromal stiffness; increased resistance to ectatic deformation	Proven efficacy in slowing or halting disease progression, with long-term stabilization reported up to 10 years	Limited corneal reshaping; protocol-dependent efficacy; possible complications include haze, infection, and delayed epithelial healing	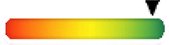 Very high: direct stromal stiffening	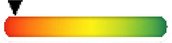 Very low: no direct reshaping
	Intracorneal Ring Segment (ICRS)/Corneal Allogenic Intrastromal Ring Segment (CAIRS)	Stromal lamellar separation with arc shortening effect leading to central cornea flattening	Redistribution of internal stresses, with corneal flattening mainly driven by geometric remodeling rather than intrinsic tissue stiffening	Improvement in corneal shape and visual acuity	Variable outcomes; empirical nomograms; limited predictability; minimal intrinsic stromal stiffening except possibly with continuous designs; risk of migration or extrusion	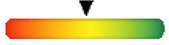 Moderate: structural support	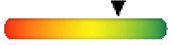 High: corneal flattening
	Combined ICRS + CXL	Combination of implant-induced geometric remodeling with CXL-induced stromal stiffening	Potentially synergistic effect of structural reshaping by ICRS and increased stromal rigidity by CXL	Enhanced biomechanical stability through combined corneal reshaping and reduction of ectatic progression	Optimal timing and sequencing not standardized; variable patient-specific response	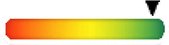 Very high: combined stiffening and support	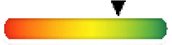 High: corneal flattening
Emerging approaches	Gene Therapy/Cell-Based Approaches	Targeted modulation of disease-related pathways and/or restoration of the keratocyte function	Potential restoration of stromal homeostasis and ECM organization	Potential modulation of ECM turnover and tissue repair; visual improvement remains unproven clinically	Experimental stage; lack of robust clinical evidence	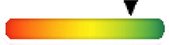 High: potential stromal stiffening through tissue remodeling	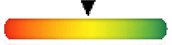 Moderate: potential geometrical reshaping
	Stromal Lenticule Implantation	Intrastromal implantation of a lenticule to increase stromal thickness and modify corneal curvature	Direct structural reinforcement through tissue addition; improved stress distribution	Increased corneal thickness with variable or moderate corneal flattening	Limited long-term data; surgical complexity; donor tissue variability.	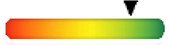 High: potential tissue reinforcement	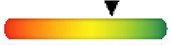 High: potential corneal flattening
	Tissue-Engineered Scaffolds	Replacement or reinforcement of stromal tissue using biosynthetic scaffolds that mimic the corneal ECM	Structural support with potential biological integration and ECM-like stromal remodeling	Corneal re-epithelialization; stromal thickness restoration	Early clinical stage; biomaterial optimization required; variability in integration and long-term stability	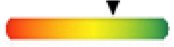 High: potential stromal stiffening through tissue remodeling	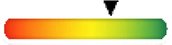 High: potential corneal flattening

CXL, corneal crosslinking; ECM, extracellular matrix; ICRS, intracorneal ring segments. The red-to-green scale qualitatively indicates the relative contribution of each strategy to biomechanical reinforcement or geometric modification. Biomechanical reinforcement, geometric modification, visual improvement, and disease stabilization are distinct outcomes. Biomechanical reinforcement refers to intrinsic or structural resistance to deformation; geometrical modification refers to changes in corneal shape; clinical outcomes refer mainly to visual or refractive improvement; and disease stabilization requires longitudinal evidence of slowed or arrested progression. The ratings represent a synthesis based on the dominant mechanism of action, the available experimental or biomechanical data, and the available clinical evidence. Low indicates absent, limited, or temporary contribution; moderate indicates partial or indirect contribution; high indicates a direct or more established contribution. For emerging approaches, the ratings reflect proposed or potential effects, as clinical evidence remains limited.

While recent reviews have thoroughly investigated keratoconus treatment strategies and their clinical outcomes, less focus has been placed on comparing these approaches according to their biomechanical effects. This mini-review summarizes current strategies aimed at stabilizing the keratoconic cornea from a biomechanical perspective, distinguishing external optical/mechanical support, intrinsic stromal reinforcement, and stromal implants that redistribute corneal stress and reshape the keratoconic cornea. Throughout this review, corneal stiffening, geometric flattening, visual improvement, and disease stabilization are treated as distinct outcomes. Stiffening reflects increased resistance to deformation, flattening reflects changes in corneal shape, visual improvement reflects functional benefit, and stabilization requires longitudinal evidence of slowed or arrested progression. Therefore, geometric or visual improvement should not be considered equivalent to true stabilization.

This narrative mini-review was based on a targeted literature search conducted primarily through PubMed. Search terms included combinations of “keratoconus”, “corneal biomechanics,” and keywords related to the principal therapeutic approaches for keratoconus. Additional treatment-specific terms were combined with biomechanical keywords to identify studies addressing the effects of each intervention on corneal mechanical behavior.

## 2 Optical rehabilitation and external biomechanical support with conservative treatments

In the early stages of KCN, visual acuity can often be corrected with spectacles; however, as the disease progresses, their effectiveness declines and CLs become the primary option for visual rehabilitation (15). Fitting CLs in ectatic corneas is challenging and depends on disease severity, cone location, and patient tolerance (16). The mainstay of visual correction is represented by rigid gas permeable (RGP) CLs, typically manufactured from combinations of silicone and acrylate-based materials (17). Their rigid surface enables the formation of a tear lens between the contact lens and the irregular cornea, which neutralizes a large portion of the corneal irregular astigmatism (18). However, the high rigidity of RGP CLs (Young's modulus in the range 1 - 1.9 MPa) (19–21), has been associated with reduced contact area with the cornea, high contact pressure, and poor centration and conformity in highly irregular corneas (22). Excessive contact pressure remains a critical factor that may lead to discomfort, epithelial damage, corneal deformation (22), disruption of the tear film and high frictional shear stress on the corneal surface (21, 23). To mitigate these issues, soft contact lenses based on hydrogel materials have been considered (24). Nevertheless, their tendency to drape over the irregular corneal surface often limits visual improvement (25). Furthermore, their lower stiffness (Young's modulus 0.4 to 0.6 MPa) (20) can make handling more difficult and may reduce adequate lens movement on the eye, thereby limiting effective tear exchange beneath the lens (26). Alternative lens designs have been developed for patients with poor tolerance or inadequate fitting of conventional RGP CLs. Hybrid lenses combine a rigid central zone with a soft peripheral skirt to preserve the optical benefits of RGP CLs while improving centration and comfort (27), whereas piggyback systems place a rigid CL over a soft silicone-hydrogel lens (28, 29). In more advanced or irregular KCN, scleral lenses may be considered, as they vault the cornea and limbus, creating a fluid reservoir that masks irregular astigmatism and reduces direct corneal contact (30–34). However, limited tear exchange may lead to complications such as midday fogging, epithelial bogging, and inflammatory responses (35).

Overall, CLs primarily improve visual function by providing optical correction and, depending on lens design and fitting, may offer external structural support (34). However, they do not intrinsically reinforce the ectatic stroma or modify the underlying ectatic process. If improperly fitted, CLs may induce epithelial injury, discomfort, disruption of the tear film, or other corneal surface complications, which may ultimately contribute to worsening of the condition (28, 35). Thus, CLs should be regarded primarily for the management of KCN symptoms, underscoring the need for additional strategies aimed at biomechanical reinforcement of the cornea.

## 3 Intrinsic stromal biomechanical reinforcement: corneal crosslinking (CXL)

Corneal biomechanical stability is primarily governed by the molecular structure of collagen and its spatial organization within the stromal matrix (36). On this principle, CXL aims to stabilize the ectatic cornea by inducing the formation of additional covalent bonds between collagen fibrils via riboflavin-mediated stromal photoactivation with ultraviolet-A (UV-A) irradiation, enhancing corneal stiffness and biomechanical integrity (37) and thereby slowing or halting the progression of KCN. CXL can be performed with epithelial removal (epithelium-off, “epi-off”) to maximize stromal riboflavin penetration, or with the epithelium left intact (transepithelial, “epi-on”) to reduce postoperative pain and complications. These two approaches are discussed in the followings.

The standard “epi-off” approach, known as the Dresden protocol, involves removal of the corneal epithelium, followed by riboflavin saturation and UV-A irradiation at 370 nm with an irradiance of 3 mW/cm² for 30 min, delivering a total fluence of 5.4 J/cm² (38). This process generates reactive oxygen species, which induce covalent bond formation between collagen fibrils, resulting in increased corneal rigidity and resistance to ectatic deformation (13)**.** The biomechanical effect of standard epi-off CXL has been demonstrated in both experimental and clinical settings. *Ex vivo* stress–strain testing showed an approximately 4.5-fold increase in corneal rigidity (Young's modulus increase from 1.3 MPa to 5.9 MPa) (39). *In vivo* Corvis ST measurements suggest a possible increase in biomechanical resistance after CXL, with stiffness parameter at first applanation (SP-A1), an indicator of the resistance of the cornea to deformation, increasing from 75.7 mmHg/mm to 77.5 mmHg/mm (40). However, SP-A1 is influenced by intraocular pressure and corneal thickness and should therefore be interpreted cautiously. The efficacy of the standard epi-off technique in stopping the progression of KCN is well established by both short- and long-term studies, with sustained stabilization of KCN and maintained improvements in clinical outcomes reported for up to 10 years of follow-up (41–43).

Several modifications of standard CXL have been introduced, including accelerated protocols that increase UV-A irradiance while reducing exposure time without altering the total energy delivered (44)**,** or protocols with pulsed UV-A delivery to enhance stromal oxygen availability during irradiation, thereby potentially augmenting the biomechanical stiffening effect (7). Direct comparisons among CXL protocols should be interpreted cautiously, as available studies differ in experimental model, measurement method, oxygen availability during irradiation, and follow-up duration that may influence the reported treatment effects.

Transepithelial CXL (T-CXL) is performed without epithelial removal (“epi-on”) to accelerate recovery and reduce pain and complications associated with debridement, including infectious keratitis (45). However, tight epithelial junctions limit stromal riboflavin diffusion during the procedure, potentially limiting its effectiveness in the biomechanical reinforcement of the cornea with respect to the standard epi-off technique (46). In this regard, *in vivo* studies in rabbit corneas have shown that Young's modulus increased by approximately 21.3% following epi-on CXL (from 9.81 MPa to 11.9 MPa), compared with an increase of approximately 102.5% after standard epi-off CXL (from 9.91 MPa to 19.86 MPa) (47). This observation may also explain the inferior impact on limiting corneal ectasia progression of epi-on vs. epi-off CXL (48, 49).

Iontophoresis-assisted CXL (I-CXL) has been proposed as an alternative approach to enhance riboflavin penetration into intact corneal tissue by means of a low-intensity electric current applied to a negatively charged, low-molecular weight riboflavin solution (46, 50)**.** However, stress–strain analysis performed through uniaxial tensile testing of corneal strips demonstrated that I-CXL significantly increases corneal stiffness in terms of Young's modulus relative to untreated controls, although to a lesser extent than standard CXL (I-CXL: 17.2 ± 8.2 MPa; standard CXL: 18.8 ± 7.2 MPa; untreated control: 8.6 ± 3.5 MPa) (51). As a consequence, its corneal flattening effect is less pronounced than that observed with standard CXL, with a 2-year mean keratometry (Kmean) reduction of 2.1% for I-CXL compared to 4.3% for standard CXL (52). Although the primary therapeutic goal of CXL is to arrest ectatic progression through stromal stiffening, corneal flattening may occur as a secondary outcome (53–55). This effect is less predictable than stabilization and depends on disease stage, cone location, corneal thickness, epithelial remodeling, and treatment protocol. Customized CXL protocols have been investigated to modulate this biomechanical response by delivering spatially selective UV-A irradiation based on individual corneal topography (53, 54). In a prospective study, a flattening of ≥2.0 D in 37% of eyes treated with personalized CXL compared to 11% after conventional CXL (*P* = 0.03) was reported, suggesting a more pronounced flattening effect with personalized irradiation patterns (53), while patient-specific computational models are emerging to predict CXL-induced corneal shape changes and guide individualized treatment planning (55). Overall, standard (epi-off) CXL remains the reference solution for progressive KCN, whereas T-CXL and I-CXL protocols offer improved tolerability but generally show weaker or less consistent biomechanical effects (47, 51). In thin corneas, standard CXL is limited by endothelial safety considerations. Modified approaches, including hypo-osmolar riboflavin swelling (56), contact lens-assisted CXL (57), individualized fluence protocols such as Sub400 (13), and stromal expansion with refractive lenticules (58) have been proposed to extend biomechanical reinforcement to thin ectatic corneas. In particular, clinical evidence for contact lens-assisted approaches is expanding. A recent retrospective study evaluating accelerated contact lens-assisted CXL using a UV-filter-free Nelfilcon A daily hydrogel lens in thin corneas reported improved best-corrected visual acuity, modest keratometric flattening, stable endothelial cell counts, and only transient clinically insignificant haze during 6 months of follow-up (59). However, the efficacy of these modified protocols in providing biomechanical reinforcement and their long-term comparability with standard epi-off CXL remain areas of active investigation.

## 4 Biomechanical reinforcement with implants: synthetic and allogenic ring segments

Implants can be broadly divided into synthetic PMMA ring segments (ICRS) and biologic allogenic corneal tissue segments (CAIRS). They are inserted with a minimally invasive procedure into the deep corneal stroma, within stromal tunnels or a corneal pocket. Unlike CXL, which primarily increases stromal material stiffness, these implants act as spacer elements between stromal collagen lamellae, inducing an arc-shortening effect that flattens the central area of the cornea (60–64). Computational biomechanical models based on finite element analysis have shown that the flattening effect observed after ICRS implantation is directly proportional to the thickness of the segment and inversely proportional to the corneal diameter (65). Additionally, ICRS implantation induces stretch in the strain-stiffening collagen lamellae at the corneal periphery that increases the effective structural stiffness, contributing to limit corneal protrusion (66). However, the main measurable effect of ICRS/CAIRS is geometric remodeling, whereas their contribution to long-term disease stabilization remains supported by limited evidence and depends on whether such increase in effective structural stiffness is sufficient to reduce further ectatic deformation. Clinical decision-making is guided by implantation nomograms, which define parameters such as number of segments, arc length, thickness, and implantation site. However, these nomograms are largely empirical, often based on unpublished clinical data, and lack correspondence with predictions of corneal biomechanical response (62). A variety of ICRS designs with different geometries, diameters, and thicknesses are currently available, including Intacs, KeraRing, Ferrara Ring, MyoRing, and more recently the allogenic option CAIRS (67).

The main characteristics of ICRSs are summarized in Table 2. In detail, Intacs (Addition Technology Inc, Des Plaines, IL, USA), KeraRing (Mediphacos Belo Horizonte, Brazil), and Ferrara segments (Mediphacos Belo Horizonte, Brazil) are crescent-shaped PMMA implants inserted into stromal tunnels to induce corneal flattening through peripheral tissue addition and arc-shortening (68, 69). Differences in cross-sectional geometry, arc length, thickness, and implantation diameter modulate the flattening effect and visual outcome (Table 2) (65). Clinical studies generally report reductions in keratometry and improvements in corrected visual acuity (Table 2), although the magnitude of response varies according to cone morphology, disease severity, tunnel depth, segment design, and nomogram selection (70–72). Importantly, available biomechanical data suggest that synthetic ICRS mainly provide structural support rather than directly increasing intrinsic stromal stiffness.

**Table 2:** Comparative characteristics of currently available synthetic and allogenic intrastromal ring implants for keratoconus (intracorneal ring segments, ICRS, and corneal allogenic intrastromal ring segments, CAIRS, respectively).

	Cross-sectional Shape	Arc Length	Available Thicknesses (*μ*m)	Indications	Reported Flattening Effect	Complications
Intacs	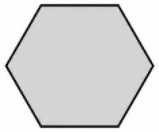 Hexagonal (68)	150° (68)	250 to 450 (68)	Mild-to-moderate keratoconus (Kmax <58 D), minimum corneal thickness ≥400 μm, central or paracentral cones (100)	Kmax reduction of approximately 3.8 D in 3 years (70)	Extrusion in 0.4% of cases, associated with trauma (70)
KeraRing	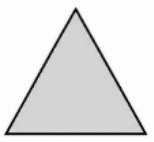 Triangular (101)	90°, 160°, 210° (101)	150 to 300 (101)	Moderate-to-advanced keratoconus (46 ≤ Kmax <65 D) with minimum corneal thickness ≥400 μm (102)	Kmean reduction of 2.24 D at 6 months (71); Kmean decreased from 50.63 ± 3.97 D to 47.56 ± 4.46 D at 24 months (73)	Early segment migration in ∼6% of eyes; no extrusion reported (71, 103)
Ferrara	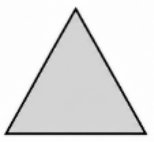 Triangular (104)	90°, 120°, 140°, 160°, 210° (104)	150 to 300 (104)	Progressive keratoconus, increasing corneal steepening, and contact lens intolerance. Contraindicated in advanced disease with apical scarring and keratometry >60 D (104)	Kmax decreased from 54.07 D to 48.09 D at 5 years (72); from 56.8 D to 54.4 D in another 5-year study (105)	Extrusion (4.8%), repositioning/exchange (12.9%), visual disturbances (∼10%), disease progression (26%) (100, 105)
MyoRing	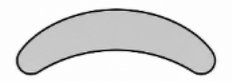 Convex to concave (106)	360° (106)	∼200 to 400 (106)	Keratoconus with contact lens intolerance, Kmax 42–65 D, and central pachymetry ≤400 μm (75)	Kmean decreased from 49.85 D to 47.51 D (−2.34 D) at 3 years (107); greater Kmax reduction than KeraRing (−7.75 D vs −4.62 D) (75)	No major complications reported; occasional need for additional interventions due to patient dissatisfaction (107)
CAIRS	Not standardized: allogenic corneal stromal tissue; customized uniform, tapered, or asymmetric segments.	Not standardized; variable/customized; reported values of approximately 160° (108, 109).	Not standardized; variable/customized.	Moderate-to-advanced keratoconus (78) with minimum corneal thickness 350 μm (110)- 400 μm (109).	Kmax reduction from 55.85 ± 7.53 D to 50.69 ± 6.38 D at 1 year (78).	No major complications reported in early series; possible segment displacement/repositioning; long-term safety data remain limited (76, 77).

KCN, keratoconus; Kmax, maximum keratometry; Kmean, mean keratometry.

The continuous 360° configuration of MyoRing (DIOPTEX GmBh, Linz, Austria) provides a circumferential support effect that differs from segmental crescent-shaped ICRSs. Unlike segmental ICRS, which are implanted in a stromal tunnel, MyoRing is inserted into a corneal pocket (73). Biomechanically, the implant acts as an artificial limbus, redistributing corneal load around the entire corneal periphery and inducing more symmetric central flattening than segmental ICRSs. This effect has been associated with an increase in effective corneal stiffness; however, the magnitude of this increase may depend on the measurement approach used (73, 74). Comparative studies suggest a greater reduction in maximum keratometry (Kmax) with MyoRing (from 51.46 ± 4.38 D to 43.71 ± 1.57 D) compared to KeraRing (from 49.66 ± 3.73 D to 45.04 ± 2.0 D) (75). However, the evidence base remains smaller than for conventional ICRS, and additional comparative studies are needed.

CAIRS may represent a shift from inert mechanical spacing toward biologically integrated stromal reinforcement, but its long-term stabilizing effect and comparative performance against synthetic ICRS require further validation. CAIRS are derived from donor corneal stromal tissue and implanted into stromal channels (76). Their mechanism relies on stromal integration and corneal reshaping through tissue addition, making it compatible also for thinner corneas. Furthermore, CAIRS can be customized in size and shape to better match individual corneal anatomy and ectasia patterns. However, this technique remains relatively recent, with limited long-term clinical data available (77). Additionally, unlike synthetic ICRS, there is no universally accepted nomogram, and the possibility of adjusting parameters such as tunnel depth and width increases procedural complexity (78).

As intrastromal implants and CXL act through different biomechanical mechanisms, combination strategies have been increasingly explored. ICRS or CAIRS can regularize corneal geometry and redistribute stromal stress, whereas CXL can increase stromal material stiffness and reduce the risk of further ectatic progression (79, 80). The theoretical advantage is therefore complementary: reshaping plus biomechanical reinforcement. Available comparative clinical evidence suggests a possible benefit of combined treatment. In a retrospective nonrandomized comparative case series, the addition of CXL to inferior-segment ICRSs implantation resulted in greater functional and topographic improvements compared with ICRSs alone. Specifically, the combined treatment group demonstrated a larger reduction in Kmean (−1.34 ± 1.27 D) versus the ICRS-only group (−0.64 ± 2.40 D). The higher best-corrected visual acuity gain (6.5 lines vs. 0.93 lines) suggests a functional advantage associated with the combined approach (81). Future studies should clarify whether combined procedures produce additive biomechanical stabilization, beyond topographic flattening and refractive benefit.

## 5 Current challenges and future perspectives

Although current therapeutic strategies can provide external support, increase stromal stiffness, or remodel corneal geometry, important challenges remain, particularly in advanced keratoconus. These include tolerability, limited predictability of refractive outcomes, and uncertain long-term biomechanical stability. Accordingly, current research is increasingly directed toward strategies that aim not only to stabilize the ectatic cornea, but also to restore stromal thickness, extracellular matrix organization, and corneal function (82, 83).

Among these approaches, cell-based therapies aim at re-establishing the anatomical integrity and physiological function of the keratoconic cornea through restoration of the keratocyte population, i.e., the principal stromal cells (84–86). Corneal stromal stem cells and adipose-derived adult stem cells (ADASCs) have been investigated as potential sources. Early clinical studies using autologous ADASCs (85), decellularized stromal laminas, or ADASC-recellularized laminas (87) reported favorable safety profiles but moderate improvements in visual and pachymetric outcomes (88). However, these findings remain preliminary, with limited direct assessment of corneal biomechanical properties. Complementary strategies such as gene- and growth factor–based approaches aim to modulate molecular pathways involved in stromal cell activity and extracellular matrix turnover. In principle, these strategies could promote controlled tissue repair and matrix reorganization, thereby restoring stromal homeostasis and biomechanical stability of the keratoconic cornea (85). However, their application to keratoconus remains mainly preclinical, and clinical evidence demonstrating biomechanical stabilization is currently lacking.

Additional strategies seek to replace or reinforce diseased stromal tissues. Stromal lenticule addition techniques aim to restore stromal volume by implanting donor or refractive lenticules within a stromal pocket. From a biomechanical perspective, these procedures may reinforce the ectatic cornea by increasing tissue thickness, modifying corneal curvature, and improving stress distribution (89). When combined with CXL, lenticule implantation combines tissue addition with stromal stiffening. Early clinical studies have reported increased corneal thickness, corneal flattening, and favorable short-term safety profiles (90), but the evidence remains limited by small cohorts and short follow-up, with reports of persistent interface haze beyond 12 months postoperatively (91). Alternatively, tissue-engineering strategies use synthetic, biological, or hybrid scaffolds designed to mimic the corneal extracellular matrix. Synthetic polymers such as polylactic acid, polyglycolic acid or poly(lactic-co-glycolic) acid offer tunable mechanical and degradation properties, whereas biologically derived matrices composed of extracellular components (e.g., collagen, laminin, or chitosan) may provide greater bioactivity and cellular compatibility (85). Collagen-based scaffolds are particularly relevant because collagen is the principal structural component of the corneal stroma, constituting approximately 70% of the corneal extracellular matrix (89), and a major determinant of transparency and biomechanical integrity (89, 92). Early clinical studies of biosynthetic corneal substitutes have shown stromal integration, re-epithelialization, and preservation of corneal transparency in selected patients (93). Nevertheless, their application to keratoconus remains at an early stage, with challenges related to optical transparency, biomechanical strength, degradation behavior, host integration, epithelial healing, and long-term stability.

Emerging therapies for keratoconus converge on two complementary biomechanical goals: restoration of stromal tissue and modulation of biological remodeling. Tissue-addition strategies, such as lenticule implantation and tissue-engineered scaffolds, primarily aim to modify the corneal geometry by increasing stromal volume. Alternatively, cell-based and molecular approaches seek to restore stromal homeostasis and extracellular matrix organization. These approaches suggest a shift toward combined structural, biological, and biomechanical restoration. However, they remain investigational, and their role in biomechanical stabilization will require controlled studies with longer follow-up and standardized biomechanical endpoints capable of quantifying the structural reinforcement of the ectatic cornea. In this sense, future studies investigating novel therapeutic strategies for ectatic corneas should complement conventional clinical outcomes, such as corneal thickness, curvature, and visual acuity, with objective and standardized biomechanical measurements. Quantifications based on, for example, stromal stiffness, corneal hysteresis (94), Brillouin microscopy (95), optical coherence elastography (96), or patient-specific computational modeling (97), would help determine whether a novel therapeutic approach truly reinforces the ectatic cornea, rather than merely improving its thickness or shape.

In conclusion, the therapeutic landscape for keratoconus has progressively evolved from purely refractive compensation toward strategies explicitly targeting corneal biomechanics. Future developments delineate a transition toward a comprehensive, multimodal therapeutic strategy in which biomechanical stabilization, corneal remodeling, and biological regeneration are integrated according to disease stage and individual corneal behavior. In this context, the digital twin paradigm may represent a promising framework, allowing patient-specific corneal models to integrate tomographic, biomechanical, and clinical data to simulate disease progression, predict treatment outcomes, and support individualized planning of the therapeutic strategy (98, 99). Ultimately, future keratoconus management should increasingly incorporate a biomechanical perspective, with interventions designed to modify the mechanical behavior of the ectatic cornea, arrest progression, and restore functional corneal integrity.
